# Genetic Relationships of Ethnic Minorities in Southwest China Revealed by Microsatellite Markers

**DOI:** 10.1371/journal.pone.0009895

**Published:** 2010-03-29

**Authors:** Hongbin Lin, Hao Fan, Feng Zhang, Xiaoqin Huang, Keqin Lin, Lei Shi, Songnian Hu, Jiayou Chu, Duen-Mei Wang

**Affiliations:** 1 CAS Key Laboratory of Genome Sciences and Information, Beijing Institute of Genomics, Chinese Academy of Sciences, Beijing, China; 2 Institute of Medical Biology, Chinese Academy of Medical Sciences, Kunming, China; 3 Graduate University of Chinese Academy of Sciences, Beijing, China; 4 Biochemistry Department, Kunming Medical College, Kunming, China; Max Planck Institute for Evolutionary Anthropology, Germany

## Abstract

Population migrations in Southwest and South China have played an important role in the formation of East Asian populations and led to a high degree of cultural diversity among ethnic minorities living in these areas. To explore the genetic relationships of these ethnic minorities, we systematically surveyed the variation of 10 autosomal STR markers of 1,538 individuals from 30 populations of 25 ethnic minorities, of which the majority were chosen from Southwest China, especially Yunnan Province. With genotyped data of the markers, we constructed phylogenies of these populations with both *D_A_* and *D_C_* measures and performed a principal component analysis, as well as a clustering analysis by *structure*. Results showed that we successfully recovered the genetic structure of analyzed populations formed by historical migrations. Aggregation patterns of these populations accord well with their linguistic affiliations, suggesting that deciphering of genetic relationships does in fact offer clues for study of ethnic differentiation.

## Introduction

Other than the majority Han Chinese, there are 55 ethnic minorities living in China, composing 9.44% of the Chinese national population (2006 data from the National Bureau of Statistics of China). Most of these minorities inhabit peripheral regions of China, especially border provinces such as Yunnan, Guangxi, and Tibet, where special landforms like the Hengduan Mountains vastly influenced their lives and history [1]. These minorities occupy their individual indigenous homelands where native mythologies are disseminated, following distinctive local traditions as they go about their daily lives. Such diversity has long caught the interest of researchers in ethnology [1], anthropology [2], [3], linguistics [4], and population genetics [5], [6], [7].

Recent researches on East Asian populations have benefited from STR markers [7], Y chromosome bi-allelic markers [8], [9], and mtDNA variations [10], [11]. These studies showed that East Asians originated in Africa and then migrated into East Asia tens of thousands of years ago. Additionally, researchers constantly observed distinct genetic divergence between northern and southern Chinese populations. Some researchers proposed a relatively recent “southern origin” of modern humans (in East Asia) via an entry from Southeast Asia followed by a northward migration [7], [9]. Others argued that this kind of divergence might result only from isolation by distance [12]. Still others proposed a north/west origin of certain haplogroups [13]. Irrespective of what the proper explanation is, Southwest China played an important role either as an entrance of migration from Southeast Asia or at least as an interface of ethnic amalgamation. As migration is the basic source of ethnic formation and differentiation [2], investigations of genetic relationships of populations resulting from migration are of enormous help in understanding the history of ethnic differentiation and today's high-degree ethnic diversity in China.

In addition, close relationship between language and nationality has long been observed [5], [14], [15]. Most ethnic minorities living in Southwest China have diverse languages that are phonologically and grammatically different from Chinese. Various branches of the Tibeto-Burman, the Tai-Kadai and the Mon-Khmer languages prevail in this relatively small region [4]. Analysis of relationship between such diversity of languages and genetic variation of populations can facilitate both ethnology and anthropology researches.

Microsatellite markers have been broadly used for analyzing relationships between human populations [16], [17], [18], [19], [20], as well as those of populations of other species [21], [22], [23]. Their abundant presence in genomes, high mutation rates, and multi-allelic nature [24] make such markers among the best choices for analysis of continental and even regional level questions of population genetics [25], [26]. Uniparentally transmitted markers like those of the non-recombinant Y chromosome and mtDNA were not chosen for this study because we wanted to analyze the information freely flowing in both male and female samples.

In this study, we surveyed the variation of 10 STR markers dispersed on Chromosome 3 for a total of 1,538 individuals in 30 populations, most of which come from southwestern provinces of China. Through analysis of the variations, we assessed the genetic diversity and interrelationships of these populations and tried to evaluate the reciprocal influences of languages and genetic structure among populations mostly living in contiguous regions.

## Materials and Methods

### Sampled populations and DNA preparation

**Figure 1:**
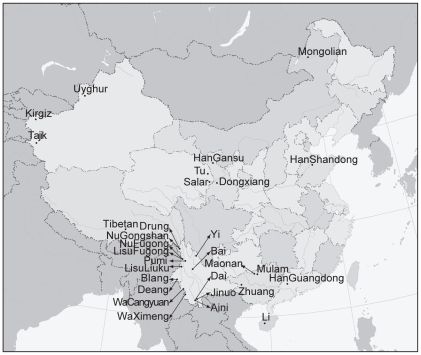
Geographical location of the 30 sampled populations.

30 populations of 25 ethnic minorities from 9 provinces of China were surveyed in this study (Figure 1, Table S1). Among these populations, 15 were from Yunnan, 3 from Guangxi, 3 from Xinjiang, and the remaining 9 were from 6 other provinces. Three Han Chinese populations from the provinces of Gansu, Shandong, and Guangdong were chosen to represent Han Chinese of Northwest China, Northeast China, and South China, respectively. Sample sizes of populations varied from 37 (Tu) to 95 (Zhuang), with the median being 50.

All 1,538 DNA samples used in this study were obtained from 30 ethnic panels of immortalized cell lines created by the Chinese Human Genome Diversity Project (CHGDP) [27], [28]. Written informed consent had been signed for the establishment of cell lines as well as subsequent studies, and this project was approved by the Ethics Committee at Chinese Academy of Medical Sciences and Peking Union Medical College.

### Genotyping and size call of alleles

Ten microsatellite markers on Chromosome 3 included in ABI Prism Linkage Mapping Set (v2.5) were selected to be genotyped. They are D3S1297, D3S1304, D3S1263, D3S1266, D3S1285, D3S1278, D3S1292, D3S1279, D3S1614, and D3S1580 (See Table 1 for genetic-map locations). These markers were chosen mainly in consideration of their heterozygosities (Table 1) documented in the ABI panel guide, since expected heterozygosity can serve as a decent proxy of informativeness of these markers [25], [29] and using more informative markers can decrease the number of markers required to be genotyped [25], [29], [30]. Although the reference heterozygosities were for the CEPH population which has a European ancestry, we tried not to make the choice of loci totally random since regional heterozygosities tend to follow similar relative order to those of loci ascertained in a geographically diverse panel [25]. Distribution of the markers (Table 1) along the chromosome and sizes of amplified products, which is important for the ease of allele size calling, were also part of the consideration for choosing loci. Mean genetic distance of adjacent markers is 22.2 cM, with the minimum being 8.2 cM; this means LD wouldn't be an issue for analysis of population genetics.

**Table 1:** Averaged heterozygosities (*H_E_*) for the 10 analyzed markers.

	D3S1297	D3S1304	D3S1263	D3S1266	D3S1285	D3S1278	D3S1292	D3S1279	D3S1614	D3S1580
Map Position (cM)	8.3	22.3	36.1	52.6	91.2	129.7	146.6	169.6	177.8	207.7
mean	0.720	0.798	0.883	0.702	0.709	0.759	0.871	0.767	0.750	0.825
s.d.	0.055	0.025	0.021	0.040	0.048	0.062	0.022	0.053	0.041	0.046
CEPH	0.820	0.800	0.860	0.730	0.730	0.870	0.850	0.850	0.830	0.840

CEPH data were from the panel guide of ABI Prism Linkage Mapping Set v2.5.

Dye-labelled primers from the aforementioned mapping set were 1∶10 diluted to 1pM for subsequent amplification reactions. After optimization, a 5-µL final volume with 0.5 µL PCR buffer (TaKaRa Dalian), 1.25nmole dNTPs, 12.5nmole MgCl_2_, 1 µL primer, 1520 ng DNA, and 0.25U Taq DNA polymerase (TaKaRa Dalian) was adopted to perform polymerase chain reactions (PCR). Thermal cycling on the GeneAmp PCR System 9700 (Applied Biosystems) included a 5 min denaturation at 94°C, followed by 10 cycles of 30 s at 94°C, 30 s annealing at 55°C, and 30 s extension at 72°C as well as another 25 modified cycles with a denaturation temperature of 89°C, and a final extension at 72°C for 10 min.

Electrophoresis of amplified products was conducted on an ABI 3730 XL DNA Analyzer (Applied Biosystems). For each marker, a size-call panel was trained using the software GeneMarker (SoftGenetics), with data from a random successfully typed 96-well plate. Fragment sizes of each reaction were automatically determined with established panels and then manually checked and adjusted. Output data were then readied for further analysis. Unsuccessful reactions were retried until either success or three failures.

### Analysis of genotypic data

Allele frequencies and expected heterozygosities (*H_E_*) were calculated using Arlequin version 3.11 [31]. Here we chose *H_E_* to present because it is considered a superior estimator of populational genetic variability [32]. Exact tests [33] were applied with the same software to determine departure from Hardy-Weinberg Equilibrium (HWE) for each of the 30 populations.

Previous comparison of different distance measures had shown that Nei et al's *D_A_* distance [34]

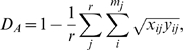
and Cavalli-Sforza and Edwards' chord distance [35]

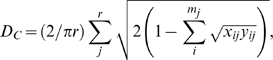
where *x_ij_* and *y_ij_* are the frequencies of the *i*th allele at the *j*th locus in population *X* and *Y*, respectively, *m_j_* is the number of alleles at the *j*th locus and *r* is the number of examined loci, are more appropriate for reconstruction of phylogenetic trees under both the infinite-allele model (IAM) and the stepwise mutation model (SMM), with or without a bottleneck effect [36]. Therefore we chose these two measures to calculate genetic distances between populations. As performance congruency of different loci ensures the legitimacy of combining markers for our following distance-based analysis [37], Mantel tests [38] were applied to pairs of distance matrices of different markers accordingly to make sure all loci behave in the same direction.

To investigate the genetic relationships of populations in a phylogenetic way, *D_A_* distances averaged over all loci were deployed to reconstruct a neighbor-joining (N-J) tree [34] with the DISPAN program [39]. Robustness of branching patterns was evaluated by a bootstrap-over-loci method with 1 000 replicates. In addition, 1 000 bootstrapped *D_C_* distance matrices by MICROSAT [40] were fed to PHYLIP [41] to construct N-J trees and ultimately generate a consensus version.

A principal component analysis (PCA) based on allele frequencies was performed in MATLAB 2007a (MathWorks Inc.) to explore the extent of correlation between genetic relationships and geographical distribution of the populations. Ahead of the analysis, frequency data were normalized for each allele by dividing the offset from mean with standard deviation. This *Z*-score process is similar to the one advocated by Cavalli-Sforza [42]. To determine the components that are truly meaningful, a parallel analysis [43], [44] was adopted. During the analysis, random datasets with the same number of variables and observations as the one being analyzed were generated and fed to PCA. Instead of comparing the scree plots of newly generated datasets with that of the original one, we used the distribution of percentages explained by the first two components in random datasets to assess the significance of components extracted in the original dataset.

In addition, the *structure* program [16] version 2.2 was used to determine a reasonable number of partitions *K* for the studied populations; clustering results were then visualized by the program CLUMPP [45]. In this clustering analysis, we assumed individuals have admixed ancestry, and that frequency distributions of different populations are correlated and thus are likely to be similar. Fifteen runs for each of *K* = 2 to 7 were carried out with both a burn-in and a run length of 50 000. The most likely *K* was then determined by comparing posterior probabilities of data under different *K* settings.

Lastly, the correlations between genetic relationship and linguistic affiliations as well as geographical distribution were assessed in a quantitative way. Linguistic distances of populations were determined according to the ‘least controversial phylogeny’ proposed by Sagart on the basis of literature [46] for phyla under consideration. In brief, the age of the most recent common ancestor (MRCA) of the Chinese and the Tibeto-Burman languages was set to 7,000 yrs BP, the MRCA age of the Mongolian and the Turkic languages set to 8,000 yrs BP, and the age of the root node, where these two MRCAs and the remaining languages were directly linked, set to 50,000 yrs BP. For instance, the linguistic distance between Drung and HanShandong was set to 7,000 yrs, and likewise the distance between Drung and Tajik set to 50,000 yrs since their languages were assumed to join these many years before present time. Geographic coordinates were determined for all populations (Table S1), and were used to compute geographic distances measured as the arc length of the great circle that passes two sampling locations. Here we did not transform the sphere distances into their logarithms, as in addition to cause non-linear distortions, the transformation may introduce infinity for population pairs that come from the same location. Correlation coefficients were calculated between above genetic (*D_A_*), geographic and linguistic distance matrices and assessed for significance by 2-way and 3-way Mantel tests [38] via permutation procedures implemented in the R package *vegan*. In addition, contributions to correlation by different linguistic groups were assessed by running Mantel tests on the data that excluded relevant populations. To assess the correlation between the distributions of populations on the PCA plot and on the Earth surface, we also ran a Mantel test for the PCA distances against the geographical distance matrix.

As previously mentioned, some PCR reactions might fail 3 times and introduce missing data. Over 90% of loci for all populations have a success rate above 80%, and most of them are greater than 90% (Figure S1). However, 5 loci in a total of 4 populations have a missing rate as high as above 60%. Under such circumstances, for listed analyses that needed to combine information from different loci, two data subsets were analyzed. The first, assigned as the full-loci dataset, contained information for all 10 loci of 26 populations – excluding Jinuo, Tibetan, WaCangyuan and WaXimeng – to maximize the bootstrap confidences of phylogenetic reconstruction. The other, assigned as the full-population dataset, contained information for all the 30 populations but of only 8 loci. D3S1304 and D3S1580 were excluded, as missing rates of the two markers for the above 4 populations were much greater than our tolerance of 40%. This latter dataset enables us to assess the positions of all studied populations.

## Results

### Genetic diversity and Hardy-Weinberg Equilibrium

To examine the diversity of selected markers, we calculated allele frequencies and expected heterozygosities (*H_E_*) for the loci of all populations (Table 1, Table S2, and Table S3). D3S1263 is the most polymorphic locus with a mean *H_E_* of 0.883 (±0.021). D3S1266 is the least polymorphic, with a mean *H_E_* of 0.702 (±0.040). The 10th and 90th percentiles of *H_E_* for all markers are 0.677 and 0.886, respectively, with the highest being 0.913 (D3S1263 of Kirgiz) and the lowest being 0.598 (D3S1266 of Dai). Mean heterozygosities of D3S1297, D3S1278, D3S1279, and D3S1614 are much lower than expected when compared to that of CEPH individuals contained in the panel guide of ABI Prism Linkage Mapping Set v2.5. Such differences are not unexpected; though, *H_E_* values indicate that selected markers are highly diversified.

Exact tests of Hardy-Weinberg Equilibrium (HWE) were applied to all markers to evaluate the extent of inbreeding within each population, given that sampling of ethnic individuals was restricted to typical habitats of corresponding populations and thus unlikely to introduce complex inner-population stratifications. Test results are summarized in Table S4. Population data of most loci are in HWE, while numbers of loci that are not in HWE vary for different populations. Marker D3S1304 and D3S1292 both include five populations that didn't pass the exact tests; the others only have one or two failures. Four in eight usable markers of the Jinuo population show departure from HWE, which suggests a sign of inbreeding in this 22,000-people ethnic group. The Dongxiang and the Salar populations also show departure from HWE at three and two loci, respectively.

### Mantel test for matrices of different loci

As a few marker-population pairs departed from HWE, Mantel tests on marker distance matrices were performed to ensure the legitimacy of joint loci analysis. Correlation coefficients and respective *P* values of each *D_A_* matrix test with the full-loci dataset are shown in Table 2. Most coefficients of marker pairs are above 0.20 and respective *P* values are all less than 0.05, suggesting distance measurements by different markers are overall positively correlated. For the 4 coefficients that are below 0.20, *P* values of 3 pairs with the marker D3S1266 are around 0.10, and that for D3S1263 with D3S1266 is as high as 0.215. Altogether, these results indicate that the performance of different marker distances is well in consistency. Therefore, it is reasonable to combine all the data for further distance-based analysis despite the existence of slight departures from HWE in our data.

**Table 2:** Mantel test results with pair of *D_A_* distance matrices of different loci.

	D3S1297	D3S1304	D3S1263	D3S1266	D3S1285	D3S1278	D3S1292	D3S1279	D3S1614	D3S1580
Map Position (cM)	8.3	22.3	36.1	52.6	91.2	129.7	146.6	169.6	177.8	207.7
D3S1297	-	0.000	0.001	*n.s.*	0.000	0.000	0.000	0.048	0.000	0.000
D3S1304	0.371	-	0.001	0.000	0.017	0.002	0.000	0.000	0.000	0.000
D3S1263	0.320	0.293	-	*n.s.*	0.000	0.006	0.000	0.039	0.015	0.000
D3S1266	0.114	0.550	0.090	-	*n.s.*	*n.s.*	0.020	0.002	0.004	0.041
D3S1285	0.616	0.306	0.388	0.119	-	0.000	0.000	0.046	0.001	0.000
D3S1278	0.601	0.352	0.285	0.121	0.661	-	0.000	0.026	0.000	0.000
D3S1292	0.421	0.419	0.413	0.290	0.593	0.493	-	0.000	0.000	0.000
D3S1279	0.208	0.537	0.218	0.542	0.227	0.301	0.467	-	0.000	0.024
D3S1614	0.426	0.482	0.242	0.422	0.509	0.497	0.502	0.441	-	0.000
D3S1580	0.581	0.444	0.403	0.246	0.680	0.696	0.532	0.294	0.511	-

Values in lower triangle are Pearson's linear correlation coefficients between each pair of matrices. Values in upper triangle are *p* values for test of coefficients based on 5000 permutations. *n.s.* stands for not significant at the 0.05 level.

### Phylogenetic reconstructions

**Figure 2:**
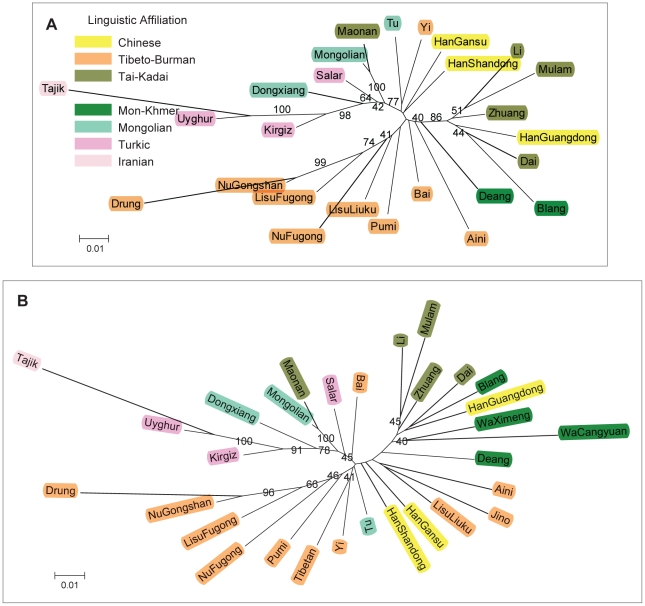
Unrooted neighbor-joining trees constructed with *D_A_* distances. (A) is the full-loci dataset and (B) the full-population dataset. Numbers labelled here represent percentage of occurrence of corresponding branches in 1,000 bootstrap replicates (where only values >40% are shown). Names of populations were coloured according to their linguistic affiliations.

Genetic relationships of the studied populations were firstly depicted by phylogenetic reconstructions. In Figure 2A, an neighbour-joining (N-J) tree built from *D_A_* matrices reveals the relationships of populations in the full-loci dataset. The populations of Tajik, Uyghur, and Kirgiz from Northwest China, together with those of Dongxiang, Salar, and Mongolian from North China compose a solid branch bearing a high bootstrap value of 77%. Within these populations, Uyghur, Kirgiz, and Salar belong to the Turkic language family, while Dongxiang, Mongolian, and Tu belong to the Mongolian language family. These two language families are all branches of the Altaic languages. Maonan, a population of the Tai-Kadai language family, also appears in this northern cluster instead of clustering with other southern populations. Repeated genotyping and examination of individual genotypes were performed for samples of the Maonan and Mongolian populations and ruled out the possibility of sample mix-up during experiment stages. Although Mongolian also appears in Yunnan as the result of military migrations and war affairs in Yuan Dynasty [1], to determine whether Maonan's abnormal position is related to such a history requires further inspections.

With several populations situated at intermediate places, the remaining ones form two distinct parts. One, including Drung, Nu, and Lisu, represents the regions of West Yunnan; the other, including Li, Mulam, Zhuang, and HanGuangdong, stands for Southeast China. Bootstrap values for the clusters of West Yunnan and Southeast China are 74% and 86%, respectively. Drung and Mulam are the innermost populations of each cluster. Similar to northern populations, southern ones also have agglomerative linguistic affiliations. The West Yunnan populations belong to the Tibeto-Burman language family of the Sino-Tibetan languages; as for the other cluster, Deang and Blang belong to the Mon-Khmer languages, whereas Li, Mulam, Zhuang, and Dai are members of the Tai-Kadai languages (previously also known as the Zhuang-Dong languages in China). Only the Maonan population, which also falls within the Tai-Kadai languages, appears at a position outside of its linguistic affiliation.

The three Han Chinese populations included in this study possess distinct positions in constructed phylogeny. HanGanshu and HanShangdong reside at the interface of the north and south clusters, as suggested by their branching sites and bootstrap values. On the contrary, HanGuangdong shows significantly close relationships to Tai-Kadai populations such as Zhuang and Dai.

In order to determine the positions for the WaCangyuan, WaXimeng, Jinuo, and Tibetan populations, another phylogenetic reconstruction was carried out using the full-population dataset, which has information of only 8 markers. As shown in Figure 2B, basic structure of the N-J tree remains the same as that of the full-loci dataset, although fewer markers yields smaller bootstrap values. The populations of WaCangyuan and WaXimeng go with the cluster of Southeast China. Tibetan falls to the group of West Yunnan. Jinuo along with Aini, of which sampling location is very close to that of Jinuo, appears at a position between the two southern clusters. Similar branching patterns were also obtained via *D_C_* matrices for both the full-loci and the full-population datasets (data not shown).

### Principal component analysis

**Figure 3:**
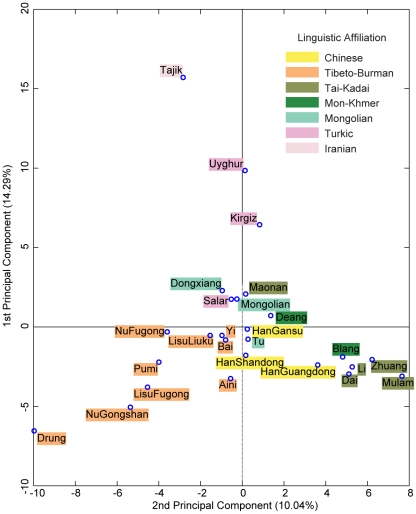
Principal component analysis with normalized allele frequencies for the full-loci dataset. Percentages of variance accounted for by the two components are indicated in labels. For better visual comparison with geographical distribution of studied populations, the plot was counter-clockwisely rotated 90°. Colouring of linguistic affiliations follows that in Figure 2a.

Besides phylogenetic reconstructions, we applied a principal component analysis (PCA) to allele frequency data of typed markers. Figure 3 is a scatter plot of the result for 26 populations along the first two components. Percentages of the overall variance accounted by the first and the second components are 14.29% and 10.04%, respectively. Parallel analysis suggests that the contributions of the first and the second components in random datasets can reach as high as 7.28 (±0.30) and 6.73 (±0.22), respectively. Among 10,000 replications, maximum contribution of the first component is 8.61%; this makes the significance of the contributions by the first and the second components in the original dataset lower than 0.0001. For the third through to the tenth components, however, the percentages gradually diminish from 6.9% to 4.0%, which are all below the contribution that can be randomly imposed by the first component (Figure S2). Therefore, the information encompassed in the first two components suggests there is statistically significant separation of studied populations. Most of the northern populations can be differentiated from those of the southern by the first component, and southern populations are further divided by the second component into southwest and southeast parts. At the same time, several populations such as Tu, Yi, Bai, and HanGanshu, have no distinct affiliations. Tajik takes the uppermost position in the scatter plot and is distant from the remaining populations. Positions of Drung and Mulam populations in the plot show evidence of extreme geographic isolations. The Altaic populations take up the upper part, and populations of the Tai-Kadai languages (except Maonan) appear in the lower right quadrant, while populations of the Tibeto-Burman language family occupy the lower left region. All these clusters of populations and linguistic affiliations resemble those in previous phylogenetic analysis. It is noteworthy that the distribution of populations in this plot well approximates their geographical locations in Figure 1, where a significant correlation (*r* = 0.418, *p* = 0.0054) of the Mantel test between PCA distances by the first two components and the geographical distance matrix can be found. Patterns revealed by PCA of the full-population dataset do not alter much; the Wa populations and the Tibetan population show affinity to the clusters of Southeast China and West Yunnan, respectively (data not shown).

### Clustering analysis by *structure*

**Figure 4:**
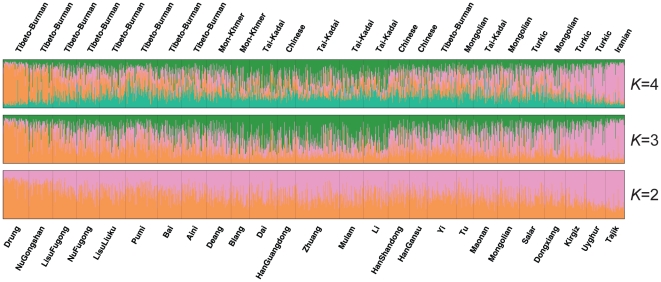
Clustering analysis by *structure* for the full-loci dataset assuming *K* = 2, 3, 4. Populations were ordered according to their respective unrooted N-J trees. Linguistic affiliations and population names are labelled above and beneath the plot, respectively. Data presented here were the results with highest posterior probabilities during 15 runs of each *K* setting.

A clustering analysis by the *structure* program was utilized to study relationships of the populations from a different point of view. The results under different *K* settings for the full-loci dataset are shown in Figure 4. Output posterior probabilities (lnPr(*X*|*K*)) from different batches suggested *K* = 3 as the most appropriate configuration according to the rules set out in the *structure* manual (Figure S3). When *K* = 2, populations such as Drung, Nu, and Lisu coming from West Yunnan and those such as Tajik and Uyghur coming from Northwest China demonstrate much higher private components (averaged member coefficients 0.22∶0.78 for Drung and 0.75∶0.25 for Tajik) than the remaining ones. This suggests that they are ordered at the innermost and the outermost locations in the regional phylogeny, which is not surprising since migrations of these Muslim populations like Tajik and Uyghur into their nowadays areas between Central Asia and East Asia happened much later than the contribution of Central Asians' ancestors to the formation of East Asian populations [1], [8] and thus are distant to the other studied populations, especially those isolated ones like Drung. At *K* = 3, the three previously identified cores, Northwest China (light purple), Southeast China (green), and West Yunnan (orange), constitute individual clusters, leaving populations like Pumi, Bai, HanShandong, HanGansu, and Tu to fall between distinct clusters. When *K* is equal or greater than 4, no new evident cluster can be introduced and proportions of this newly added part don't vary as much across most populations as those of the other three sources. Complexity of membership coefficients of populations like HanGanshu, HanShandong, Aini, Bai, and Pumi can be best illustrated by their undefined affiliations to any distinct clusters.

As a next step, *structure* analysis was applied to populations of the three cores separately to examine any additional decomposable stratification. For all three of the cores, *structure* clustering didn't yield any separation of populations within each of them (data not shown). This result was expected, as previous overall clustering had suggested that with only 10 markers employed in this study, certain level of differentiation among these populations cannot be resolved by the *structure* program. Increased number of markers might help to produce finer separation for populations of these three cores.

### Correlations between genetic, geographic and linguistic distances

As has been seen in previous phylogenetic, factor decomposition, and clustering analyses, the genetic relationships of populations shows a strong correlation with their linguistic affiliations. To explore such a correlation in a quantitative way, we statistically compared genetic, geographic and linguistic distance matrices for our population datasets (see Table S5 for detailed matrix data). Correlation coefficients and the results of two-way and three way Mantel tests between the three matrices are shown in Table 3. The correlation between genetics and linguistics (*r* = 0.239) for the full-loci dataset is slightly weaker than that between genetics and geography (*r* = 0.287). Permutation shows that the two correlations are both significant (*p* = 0.0016 and 0.038, respectively); though, the significance is greater for the former pair no matter geographical distances were controlled (in 3-way tests) or not (for 2-way tests), which is possibly because the quantification of geographical distances with arch lengths for populations in Yunnan could not take into account the effects of special landforms in that area, thus making the distances among these populations shorter than they would otherwise be. The correlation between linguistic affiliations and geography is both the highest (*r* = 0.346) and the most significant (*p* = 0.0002) among all the three pairs. Tests for the full-population dataset gave similar conclusions to the above comparisons (data not shown).

**Table 3:** Correlation coefficients among genetic (GEN), geographic (GEO), and linguistic (LING) distances.

Dataset		*r*_GEN,GEO_	*r*_GEN,LING_	*r*_LING,GEO_
Full-loci	2-way	0.287(0.038)	0.239(**0.0016**)	0.346(**0.0002**)
	3-way	0.224(0.078)	0.156(0.043)	
non-Chinese	2-way	0.286(0.048)	0.274(**<0.0002**)	0.417(**<0.0002**)
	3-way	0.196(*n.s.*)	0.177(0.024)	
non-Tibeto-Burman	2-way	0.488(**0.0002**)	0.309(**<0.0002**)	0.310(**0.0018**)
	3-way	0.434(**0.004**)	0.190(0.031)	
non-Tai-Kadai	2-way	0.270(0.065)	0.298(**0.010**)	0.416(**0.001**)
	3-way	0.168(*n.s.*)	0.212(0.048)	
non-Mon-Khmer	2-way	0.317(0.033)	0.257(**0.0006**)	0.413(**<0.0002**)
	3-way	0.239(0.060)	0.146(0.052)	
non-Mongolian	2-way	0.398(0.013)	0.274(**0.005**)	0.383(**0.001**)
	3-way	0.330(0.027)	0.144(*n.s.*)	
non-Turkic	2-way	0.304(0.087)	0.211(0.042)	0.353(**0.0012**)
	3-way	0.251(*n.s.*)	0.116(*n.s.*)	
non-Iranian	2-way	0.072(*n.s.*)	0.179(0.017)	0.316(**0.0004**)
	3-way	0.017(*n.s.*)	0.165(0.038)	

In parentheses are presented *p* values of Mantel tests by 5,000 permutations. Three-way tests were carried out by controlling the distance that does not appear in the subscript. All tests were based on the full-loci dataset, which contains information of 10 loci of 26 populations, and its derivatives by excluding populations of a specific language. Highlighted are highly significant (0.01 level) *p* values; *n.s.* stands for not significant at the 0.1 level.

When populations of a specific language were excluded from the full-loci dataset, changes in coefficients and *p* values of new tests revealed the contribution by those populations in correlation analyses. The correlation between genetic and geographical distances reduces to an insignificant (*p* = 0.253) level when Tajiks (speakers of Iranian languages) were excluded, which reminds us the special role of this population as revealed in previous PCA analysis (Figure 3). However, the correlation between genetic and linguistic distances for the same partial dataset remains to be significant (*p* = 0.017) and its coefficient is even slightly increased (*r* = 0.165) under 3-way Mantel test. Such a contrast suggests that even though the geographical dispersion may not significantly resemble the genetic relationship when Tajik was excluded, there still be a certain correlation between genetic and linguistic affiliations for these populations.

2-way Mantel tests yielded significant (*p*<0.05) correlation for all partial datasets. However, when Mongolian or Turkic populations were excluded, the coefficients between genetics and linguistics of 3-way tests for respective partial datasets reduced to an insignificant level (*p* = 0.102 and 0.176, respectively), while it is not the case for other partial datasets that excludes populations of a language like Tai-Kadai and Tibeto-Burman. This change relative to the full-loci dataset is because the exclusion of Mongolian or Turkic populations results in a reduction of averaged genetic distances among the remaining populations, and thus the correlation between genetics and linguistics with geographical distances controlled in 3-way tests is more easily confounded by regional migration events as well as the quantification method of geographical distances for closely distributed populations.

## Discussion

The effectiveness of selected markers, measured per consistency with previous investigations and historical population records, goes beyond our expectation on this study. Previous simulations have established that the number of microsatellite markers over the size of samples is important for phylogenetic study [36], [47]. In order to unravel relationships of closely related populations, around fifty markers are required to achieve a sufficient confidence level [36]. Some other studies revealed that using of highly informative markers can greatly reduce the number of markers that have to be typed while maintaining a comparable level of resolution [25], [29], [30] and such kind of informativeness is transferable to a great extent to other collections of populations [29]. Fortunately, expected heterozygosity can serve as a decent proxy of the informativeness [25], [29], [30]. In our study, we selected markers to be as polymorphic as possible, according to the information provided with the ABI Prism Linkage Mapping Set. Therefore, when higher order fine structure is not the major concern of the study and marker selection can be facilitated with prior knowledge of their diversities, much fewer than fifty microsatellite markers is satisfactory for analysis.

Scores of molecular methods and research studies have been applied to the question of peopling of East Asia [7], [8], [9], [10], [12], [13], [48]. These analyses all have confirmed the distinct makeup of populations from northern and southern regions. Although some details remain controversial [8], [9], [10], these analyses evaluated the contributions of Southeast Asians and Central Asians to the formation of East Asian populations [7], [8], [9], [10], [48]. In our work, all methods of analysis consistently support differentiation between the populations of North and South China, as well as between the Tibeto-Burman and the Tai-Kadai and Mon-Khmer populations. In a haplotype analysis of non-recombinant Y chromosome (NRY) with more markers and populations than in a previous study by Su et al. [9], Karafet et al. [8] demonstrated that Central Asians (CAS) substantially contributed to the contemporary gene pool of northern East Asians (NEAS). Another study proposed the possibility of a north/western origin in China of an NRY haplogroup [13] which has M214, but not M175, under the YCC nomenclature [49]. Rosenberg et al. [25] have shown that as a Central Asian population Uyghur can be clearly separated from typical NEAS. Thus the proximity of Uyghur and Tajik to Mongolian spoken populations observed in our study should be attributed to their ancient genetic connections that parallel with their affiliations to the Altaic languages. As for the difference between Tibeto-Burman populations and those inhabiting Southeast China, it is well in accordance with historical records that these Tibeto-Burman populations are descendants of the Di-Qiang population who emigrated from the areas of upper Yellow River to the areas surrounding the Tibetan Plateau [1]. The terrain in this new region consists of a high altitude, sheer ravines, and rip currents. All these landforms are significant barriers to frequent gene flow between populations. Thus, isolated populations like Drung and Nu all show great genetic distance to the others.

Besides differentiation of populations of different regions, gene flow and ongoing demographic processes have greatly shifted and are still shifting genetic relationships between East Asian populations [7], [8], [13]. Different analyses have consistently shown in this study that populations such as the Tu, Yi, HanGanshu, Bai, and Pumi reside at the interfaces between different clusters. This corresponds well with geographical distribution and historical records of complex migration patterns, as well as genetic intermixing of these populations [13]. For example, the migration of Han Chinese to regions of the Hengduan Mountains massively increased since the Yuan Dynasty, in response to the need of governing such regions far from central authorities based in cities such as Dadu (modern Beijing) and Nanjing. This resulted in great changes in social and political ecologies, as well as intermarriage of local populations. Besides the above-mentioned populations that fall between individual clusters, the HanGuangdong population shows distinct affiliations with southern populations (compared to the HanGanshu and HanShandong populations). Previous researches with methods such as NRY and mtDNA variations have provided evidence supporting the demic diffusion hypothesis for southward expansion of the Han culture [50]. Along with the expansion of the Han culture into southern regions, genetic composition of the migrants markedly altered as a consequence of ethnic fusion with indigenous populations.

High correlation between phylogenetic tree, or population relationships, and linguistic tree, can be created during demographic expansions [14]. Although populations of different language groups in this study are not exhaustive, agglomeration of populations in terms of linguistic affiliation is substantial in constructed phylogenies, especially for populations of the Altaic languages and those of the Tibeto-Burman language family (Figure 2). The populations of Blang and Deang as well as WaCangyuan and WaXimeng fall into the category of Mon-Khmer languages. These four populations tend to be closer than the Tibeto-Burman populations to the Tai-Kadai populations. Until today, there has been a long-term debate as to whether the Tai-Kadai languages are just a branch of the Sino-Tibetan languages or instead they should be treated as a new set [51], [52], [53]. Our results suggest speakers of Tai-Kadai languages have a closer genetic relationship to those of the Mon-Khmer languages, and therefore the Tai-Kadai languages should not be directly assigned as a sister branch of the Tibeto-Burman language family into the category of Sino-Tibetan languages. This latter conclusion fits well with the opinion of western scholars like anthropologist Paul K. Benedict [52] and linguistists Stanley Starosta [54] and Laurent Sagart [55]. The roles of populations of the Hmong-Mien languages that populate in East Yunnan and West Guangxi were not assessed in our study, and their positions in the regional phylogeny can be dissected in future finer-scale analyses.

As the peopling of East Asia is a multi-layered and multi-directional process [8], a combination of different types of markers and finer mutation models are required to detect signals of demographic events occurred at different ages. Further researches are required along these lines.

## Supporting Information

Figure S1 Distribution of percentages of missing data. The abscissa stands for the missing percentage of a specific locus of one population, and the ordinate stands for total number of loci in all populations. Only 5 loci in a total of 4 populations have a unsatisfied missing rate as high as 60%.
(0.01 MB EPS)

Figure S2 Contributions by PCA components in real and random datasets. The barplot as well as the labelled numbers stands for the contributions by the first 10 components in PCA of our real dataset. Solid line stands for the mean contributions in 10,000 random datasets and dashed lines are corresponding 95% upper bound and 5% lower bound. Contributions by the first two components, though only 24.33% in total, are much higher than that by the first component in random datasets.
(0.01 MB EPS)

Figure S3 Boxplot of posterior probabilities of the *structure* clusterings. Plotting follows conventions, where the central mark is the median, the edges of the box are the 25th and 75th percentiles, the whiskers extend to the most extreme data points not considered outliers, and outliers are plotted individually.
(0.01 MB PDF)

Table S1 Information of 30 sampled populations. Lat and Long stand for latitude (north) and longitude (east), respectively.
(0.07 MB DOC)

Table S2 Expected heterozygosities (*H_E_*) for all markers of each population. Values are crossed out for the 5 loci where missing rates within respective population are higher than 40%.
(0.02 MB XLS)

Table S3 Detailed frequency data of 10 loci for studied populations. Numbers in header line represent fragment sizes called in the GeneMarker environment. Contained in the last column of each table are the allele numbers that were successfully called for each population.
(0.09 MB XLS)

Table S4 P values of exact tests for Hardy-Weinberg equilibrium. Values<0.05 mean significant departure from equilibrium and are labeled as bold face. Values are crossed out for the 5 loci whose missing rates within respective population are higher than 40%.
(0.02 MB XLS)

Table S5 Matrices of genetic, linguistic and geographical distances used for Mantel tests. Genetic distances used here is the *D_A_* distance; linguistic distances were constructed according to the ‘least controversial phylogeny’ proposed by Sagart [44]; geographical distances were measured as the arc length of the great circle that passes two sampling locations.
(0.10 MB XLS)
