# Supplementary figures and images for: Genetic Relationships of Ethnic Minorities in Southwest China Revealed by Microsatellite Markers
Source: PLoS One. 2010 Mar 29;5(3):e9895. doi: 10.1371/journal.pone.0009895 (PMC2847899; doi:10.1371/journal.pone.0009895)

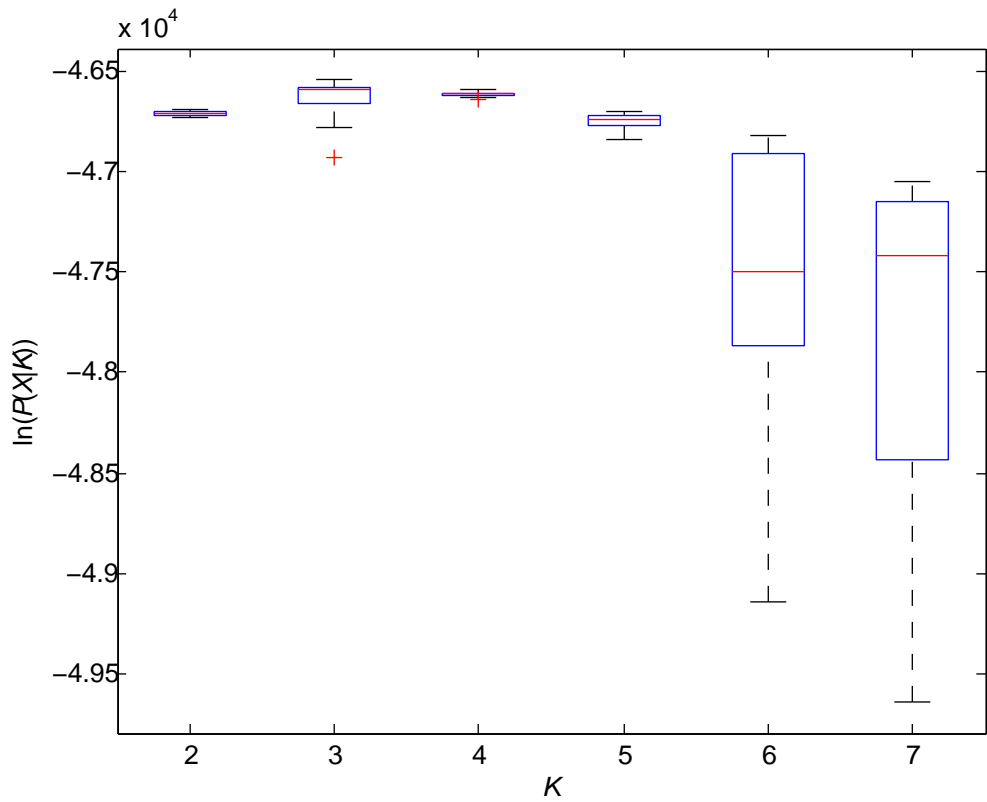

Supplement: Figure S3 — Boxplot of posterior probabilities of the structure clusterings. Plotting follows conventions, where the central mark is the median, the edges of the box are the 25th and 75th percentiles, the whiskers extend to the most extreme data points not considered outliers, and outliers are plotted individually. (0.01 MB PDF) [file pone.0009895.s003.pdf]
