# Supplementary material for: Genetic Relationships of Ethnic Minorities in Southwest China Revealed by Microsatellite Markers
Source: PLoS One. 2010 Mar 29;5(3):e9895. doi: 10.1371/journal.pone.0009895 (PMC2847899; doi:10.1371/journal.pone.0009895)
Supplement: Table S1 — Information of 30 sampled populations. Lat and Long stand for latitude (north) and longitude (east), respectively. (0.07 MB DOC) [file pone.0009895.s004.doc]

**Table S1. Information of 30 sampled populations. Lat and Long stand for latitude (north) and longitude (east), respectively.**

| Pop | # | Size | Location | Lat(N) | Long(E) | Language |
| --- | --- | --- | --- | --- | --- | --- |
| HanGansu | 22 | 39 | Wuwei, Gansu | 37.93 | 102.64 | Chinese |
| HanGuangdong | 14 | 39 | Guangning, Guangdong | 23.67 | 112.40 | Chinese |
| HanShandong | 26 | 45 | Zouping, Shandong | 36.86 | 117.74 | Chinese |
| Aini | 12 | 55 | Jinghong, Yunnan | 22.01 | 100.79 | Tibeto-Burman |
| Bai | 10 | 51 | Dali, Yunnan | 25.59 | 100.23 | Tibeto-Burman |
| Drung | 2 | 55 | Gongshan, Yunnan | 27.74 | 98.67 | Tibeto-Burman |
| Jino | 27 | 57 | Jinuoshan, Yunnan | 22.04 | 101.01 | Tibeto-Burman |
| LisuFugong | 6 | 50 | Liuku, Yunnan | 26.90 | 98.87 | Tibeto-Burman |
| LisuLiuku | 5 | 56 | Fugong, Yunnan | 25.84 | 98.85 | Tibeto-Burman |
| NuFugong | 8 | 50 | Fugong, Yunnan | 26.90 | 98.87 | Tibeto-Burman |
| NuGongshan | 9 | 52 | Gongshan, Yunnan | 27.74 | 98.67 | Tibeto-Burman |
| Pumi | 17 | 69 | Lanping, Yunnan | 26.45 | 99.42 | Tibeto-Burman |
| Tibetan | 25 | 46 | lhasa, Tibetan | 29.66 | 91.13 | Tibeto-Burman |
| Yi | 18 | 62 | Ninglang, Yunnan | 27.28 | 100.75 | Tibeto-Burman |
| Dai | 11 | 60 | Jinghong, Yunnan | 22.01 | 100.79 | Zhuang-Dong |
| Li | 1 | 53 | Baisha, Hainan | 19.23 | 109.45 | Zhuang-Dong |
| Maonan | 28 | 52 | Hechi, Guangxi | 24.83 | 108.26 | Zhuang-Dong |
| Mulam | 16 | 52 | Luocheng, Guangxi | 24.91 | 108.84 | Zhuang-Dong |
| Zhuang | 19 | 95 | Baise, Guangxi | 23.90 | 106.62 | Zhuang-Dong |
| Blang | 29 | 39 | Luxi, Yunnan | 23.44 | 98.59 | Mon-Khmer |
| Deang | 15 | 52 | Luxi, Yunnan | 23.44 | 98.59 | Mon-Khmer |
| WaCangyuan | 3 | 50 | Cangyuan, Yunnan | 23.15 | 99.25 | Mon-Khmer |
| WaXimeng | 4 | 49 | Ximeng, Yunnan | 22.64 | 99.60 | Mon-Khmer |
| Dongxiang | 23 | 45 | Dongxiang, Gansu | 35.66 | 103.39 | Mongolian |
| Mongolian | 24 | 48 | Hailaer, Inner Mongolia | 49.21 | 119.73 | Mongolian |
| Tu | 7 | 37 | Huzhu, Qinghai | 36.84 | 101.95 | Mongolian |
| Kirgiz | 20 | 45 | Wuqia, Xinjiang | 39.71 | 75.25 | Turkic |
| Salar | 13 | 53 | Xunhua, Qinghai | 35.87 | 102.43 | Turkic |
| Uyghur | 30 | 42 | Yili, Xinjiang | 43.92 | 81.32 | Turkic |
| Tajik | 21 | 40 | Tashikuergan, Xinjiang | 37.77 | 75.23 | Iranian |
